# Pt-, Rh-, Ru-, and Cu-Single-Wall Carbon Nanotubes Are Exceptional Candidates for Design of Anti-Viral Surfaces: A Theoretical Study

**DOI:** 10.3390/ijms21155211

**Published:** 2020-07-23

**Authors:** Aref Aasi, Sadegh M Aghaei, Matthew D. Moore, Balaji Panchapakesan

**Affiliations:** 1 Small Systems Laboratory, Department of Mechanical Engineering, Worcester Polytechnic Institute, Worcester, MA 01609, USA; aaasi@wpi.edu (A.A.); smaghaei@wpi.edu (S.M.A.); 2 Applied and Environmental Virology Laboratory, Department of Food Science, University of Massachusetts, Amherst, MA 01003, USA; mdmoore@foodsci.umass.edu

**Keywords:** single wall carbon nanotubes, COVID-19, copper, nickel, palladium, platinum, ruthenium, rhodium, virus, antiviral surfaces

## Abstract

As SARS-CoV-2 is spreading rapidly around the globe, adopting proper actions for confronting and protecting against this virus is an essential and unmet task. Reactive oxygen species (ROS) promoting molecules such as peroxides are detrimental to many viruses, including coronaviruses. In this paper, metal decorated single-wall carbon nanotubes (SWCNTs) were evaluated for hydrogen peroxide (H_2_O_2_) adsorption for potential use for designing viral inactivation surfaces. We employed first-principles methods based on the density functional theory (DFT) to investigate the capture of an individual H_2_O_2_ molecule on pristine and metal (Pt, Pd, Ni, Cu, Rh, or Ru) decorated SWCNTs. Although the single H_2_O_2_ molecule is weakly physisorbed on pristine SWCNT, a significant improvement on its adsorption energy was found by utilizing metal functionalized SWCNT as the adsorbent. It was revealed that Rh-SWCNT and Ru-SWCNT systems demonstrate outstanding performance for H_2_O_2_ adsorption. Furthermore, we discovered through calculations that Pt- and Cu-decorated SWNCT-H_2_O_2_ systems show high potential for filters for virus removal and inactivation with a very long shelf-life (2.2 × 10^12^ and 1.9 × 10^8^ years, respectively). The strong adsorption of metal decorated SWCNTs and the long shelf-life of these nanomaterials suggest they are exceptional candidates for designing personal protection equipment against viruses.

## 1. Introduction

The United Nations has named the SARS-CoV-2 (COVID-19) pandemic as the “most challenging” crisis since World War II [1]. COVID-19 is a disease caused by the SARS-CoV-2 virus, which is primarily thought to spread through respiratory droplets or high touch fomites. A human sneeze or cough can eject thousands of droplets carrying infectious agents (virus) [2]. Time-lapsed studies have shown that the largest droplets rapidly settle within 1 to 2 m away from the person [2]. The smaller and evaporating droplets are trapped in a turbulent puff cloud, remain suspended, and, over the course of seconds to a few minutes, can travel the dimensions of a room and land up to 6 to 8 m away [2]. Healthcare workers represent a uniquely vulnerable population, given their close proximity to infected patients (<6–8 m) in settings with abundant fomites, with further increased risk in the environments of aerosol-generating procedures (AGPs) [3]. Currently, there is no cure for the SARS-CoV-2 virus, and the only way to mitigate further spread of the disease is to wear personal protection gear (PPE) such as a mask, and in hard-hit states, there are requirements to wear one. The use of masks is primarily due to previous reports that wearing masks can decrease the amount of virus expelled through respiratory droplets—however, most cotton/paper-based masks do not entirely stop the expulsion of viruses due to size differences between viruses (mainy 30–140 nm) and cloth pore sizes (>1 micron), nor do mask materials inactivate the virus [4]. Thus, developing materials and methods to enable surfaces to capture and inactivate viruses on contact can mitigate the number of infections and lower the number of biological transmission pathways. However, the size of the viruses (30–140 nm) [5] makes it challenging to remove them physically, and the traditional cotton masks with large pore size cannot filter viruses effectively. Nanomaterials, which are the size of or smaller than a virus, can be useful for size-based capture and detection of viruses, biological inactivation, and can be loaded with drugs and disinfecting agents. Large area nanomaterial surfaces can be washed and integrated with sustainable fabrics in the form of masks and other large area surfaces for public use. However, this field is still in its infancy due to a lack of fundamental understanding of how to effectively use nanomaterials as virus inactivating surfaces.

**Figure 1:**
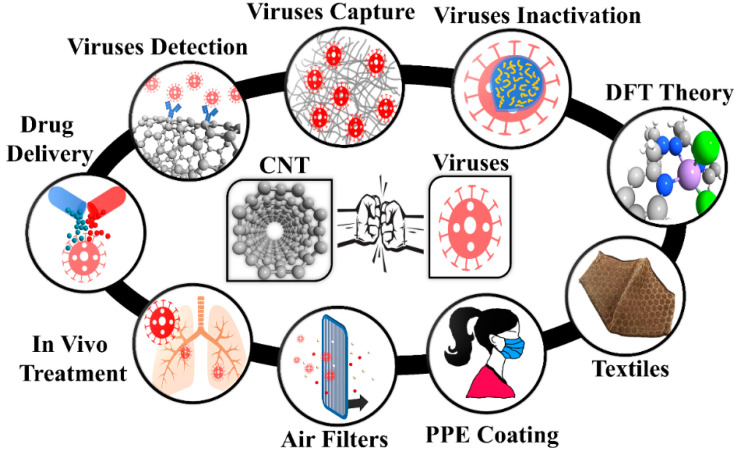
Carbon nanotubes in different segments of viral capture/inactivation. PPE: Personal Protective Equipment; CNT: Carbon nanotubes; DFT: Density Functional Theory.

Single wall carbon nanotube (SWCNTs) are recognized as a promising nanomaterial for detection, filtering, and inactivation of biological agents thanks to their high surface-volume ratio, fine pore size, low density, high mechanical strength, resistance to acids and bases, flexibility, ability to create reactive oxygen species, resistance to respiratory droplets, and biological compatibility with various drugs [6,7,8,9,10,11,12]. Figure 1 presents the potential of CNTs for the fight against viruses such as influenza and respiratory viruses such as SARS-CoV-1 and SARS-CoV-2. CNTs have been investigated as drug delivery systems [13] and for anti-HIV agents [14,15]. They have also been used to detect and capture viruses and viral proteins [16,17,18,19,20,21]. Antibody-conjugated CNTs were developed for the detection of various virus proteins [19,20]. Recently, Yang et al. claimed that the employment of acidified CNTs conjugated with special RNA lyase combined with a photothermal conversion effect has the potential to inhibit SARS-CoV-2 [21]. Ting et al. found that carbon dots combined with the antimicrobial polyphenol curcumin can significantly inhibit coronavirus entry [21]. Yeh et al. reported a size-tunable microdevice based on multiwalled CNT (MWCNT) for label-free concentration and detection of viruses (e.g., avian flu virus) [18]. The intratubular distance between MWCNTs was engineered in the 17 nm to 325 nm range to enable the size-based capture of different viruses [18]. Recently, Mycroft-West et al. reported that the SARS-CoV-2 Spike S1 protein receptor-binding domain undergoes a conformational change upon interaction with heparin [22]. Wasik et al. fabricated a chemiresistive biosensor based on the single-walled CNT (SWCNT) network functionalized with heparin for the detection of dengue virus [23]. Consequently, heparin functionalized CNTs have demonstrated the capacity to develop effective therapeutics against SARS-CoV-2.

CNT-based filters were reported to be useful for the removal of viral and bacterial pathogens [24,25,26,27,28,29,30,31,32]. Vecitis et al. showed the efficacy of MWCNT as an anodic filter for removal and inactivation of bacteria (*Escherichia coli*) and viruses (bacteriophage MS2) [28]. Nemeth et al. investigated the removal of MS2 bacteriophages from contaminated water using inorganic composite based MWCNT hybrid membranes [30]. They discovered that the MWCNT membrane coated by Cu_2_O nanoparticles removes bacteria and viruses with ≥99.99 efficiencies [30]. The investigation on the usage of MWCNTs as an airborne filter was presented by Viswanathan et al. [31]. They revealed that MWCNT-coated cellulose fiber filters show better quality for filtration compared to cellulose filters [31]. CNT based air filters can be used for various purposes, such as minimizing the transmission of viruses and sampling the bioaerosols to characterize the transmission in a hospital setting [33].

SWCNTs have also been investigated for producing breathable protective layers in fabrics. Bui et al. fabricated a composite material incorporating vertically aligned SWCNTs embedded in the polymer parylene-N [34]. The superior water flow rate in nanotube cores and their well-defined inner diameters as a high degree of protection from biothreats by size exclusion make the SWCNT-polymer composite an ultra-breathable and protective membrane [34]. As previously reported [35], nanoparticles can be used as a coating layer of the protective clothing for trapping the infectious agents. The coating of personal protective equipment (PPE) with CNTs can be performed in a similar fashion for reducing the transmission of pathogens.

It was reported that the coronavirus could be inactivated by ultraviolet light (UV) at 254 nm and heat treatment of 56 °C for 30 min [36]. Thanks to the CNT’s good photothermal conversion efficacy and superior near-infrared light absorption characteristics, CNTs can experience a sharp increase in temperature, higher than 51 °C, to realize photodynamic hyperthermia [37,38]. Hence, the infected protective CNT facemasks can be sterilized by heating, near-infrared laser irradiation, or UV radiation.

Reactive oxygen species (ROS) such as peroxide, superoxide, hydroxyl radical, singlet oxygen, and alpha-oxygen are detrimental to pathogens [39]. Warnes et al. reported the rapid inactivation of human coronavirus 229E using a range of copper alloys within a few minutes after simulated fingerprint contamination [40]. Exposure to copper destroys the viral genomes, and irreversibly affects the morphology of the virus, causing the disintegration of the viral envelope [40]. They also found that the generation of ROS on copper alloys accelerates viral inactivation [40]. Similarly, Chompoosar et al. revealed that cationic gold nanoparticles (<4 nm) could create oxidative stress that promotes the production of ROS on materials [41]. Hydrogen peroxide (H_2_O_2_), the most common peroxide, can prevent the correct functioning and replication of viruses [42,43,44]. Many living organisms intentionally produce H_2_O_2_ for several purposes, such as killing pathogens, host defense, and for cellular signaling [45,46]. Moreover, H_2_O_2_ is a strong oxidizing agent that has been widely used as a cleaning and disinfectant agent [47,48].

The capture of ROS-promoting species such as H_2_O_2_ on the CNT surfaces loaded with metal nanoparticles can serve as a dual-purpose mask for removal and inactivation of pathogens on contact. Based on the literature, carbon-based sensors are capable of H_2_O_2_ adsorption [49,50,51,52,53,54,55,56]. Doped graphene has shown much higher catalytic activity compared to pristine graphene, as obtained from experimental and theoretical studies [50,54]. Düzenli reported that the H_2_O_2_ molecule is chemically adsorbed on N-, B-, Pd-, and Pt-doped graphene surfaces [52]. Reduction of the H_2_O_2_ molecule into a H_2_O molecule and an adsorbed O atom occurs over the S-, Au-, Ag-, and Cu-doped graphene surfaces [52]. Among the various doped graphene surfaces, the most sensitive surface was determined to be the Cu-doped graphene surface, according to thermodynamic properties [52]. Silicon carbide (SiC) nanotubes were also introduced as a potential candidate for H_2_O_2_ detection [53]. The adsorption energy of a single H_2_O_2_ molecule on pristine SWCNT was found to be 0.25 eV obtained by density functional theory (DFT), suggesting that the interaction between pristine SWCNT and H_2_O_2_ is weak [56]. In order to develop a filter with a long shelf-life, it is crucial to ensure that peroxide and subsequently the virus can be retained by the CNT filter. Functionalization of SWCNTs with transition metals not only help to destroy the virus but also enhance the interactions between the sensing material and the target molecule thanks to their strong catalytic performance [57,58,59,60,61,62,63,64,65,66,67,68,69,70].

We previously reported the synthesis of randomly oriented SWCNTs and ultra-thin liquid crystal films of semiconducting SWCNTs. Also called “buckypaper”, large area surfaces (47 mm to 85 mm) of SWCNTs were formed using a simple vacuum filtration process with pore size between 100 nm to 300 nm [71,72,73]. In this study, we investigated the functionality of metal loaded CNTs promoted by peroxide to inhibit and inactivate viruses such as SARS-CoV-2. Such choices of metals on the large surface area of SWCNTs can act as a barrier for viral transmission and inactivation of viruses. To this end, we investigate the interactions of the H_2_O_2_ molecule, as a representative of ROS, on an SWCNT decorated with transition metal atoms (Pt, Pd, Ni, Cu, Rh, or Ru) using DFT to find a platform with the potential to capture and inactivate respiratory viruses such as SARS-CoV-2 and influenza. The most stable adsorption configurations, adsorption energies, charge transfer, electronic, and magnetic properties of the H_2_O_2_ on the metal-nanotube, are scrutinized. Our results encourage further experimental investigations, and it opens new doors in the development of antiviral surfaces.

## 2. Results

Figure 1 presents the schematic of how to use nanomaterials for viral capture and inactivation. There is a broad range of use of nanomaterials including capture, inactivation, theory for ROS promoting surfaces, integrating nanomaterials into textiles, large-area surfaces for mitigating transmission, size-based mitigation in air filters, drug delivery based on the favorable free energy available both inside and outside SWCNTs, and in vivo treatment such as vaccine delivery due to rich functionalization chemistry. The rich possibilities are just being realized, and we have a long way before nanomaterials can be fully utilized in the fight against viruses.

**Figure 2:**
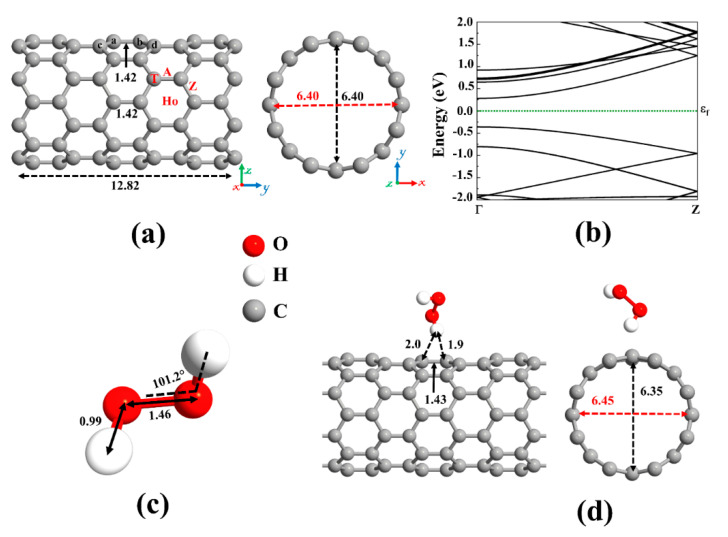
(**a**) Optimized structure of a pristine (8,0) single-wall carbon nanotube (SWCNT) and (**b**) its corresponding energy band structure, showing semiconducting behavior with an energy bandgap of 0.643 eV. The dotted green line indicates the Fermi level, which is set to zero. Different adsorption sites on an SWCNT (Ho: hollow, A: axial, Z: zigzag, and T: top). (**c**) The optimized molecular structure of Hydrogen peroxide (H_2_O_2_). (**d**) The most stable adsorption configuration of H_2_O_2_ on pristine SWCNT. Distances are given in the unit of Å. The red and black dotted arrows present the diameter of the nanotube parallel and perpendicular orientation to the axis of the nanotube, respectively.

We have focused our study on DFT based theoretical methods to enable the choice of materials for ROS promotion on SWCNT surfaces. This is important, as currently, mainly copper is known to promote significant viral inactivation (e.g., coronaviruses). To study the ROS promoting aspects of SWCNTs and its molecular composites, we first verified the accuracy of the employed computational method by optimizing the structure of the pristine (8,0) SWCNT (see Figure 2a) and calculating its energy band structure (see Figure 2b). Our results revealed that the pristine (8,0) SWCNT is a semiconductor with a direct bandgap of 0.643 eV at Γ-point. Moreover, the C-C bond length and diameter are 1.42 Å and 6.40 Å, respectively, which are in good agreement with the literature [68,74].

A carbon atom has four valence electrons. Each carbon atom in the nanotube uses its three electrons in the *sp*^2^ hybrid orbitals to create σ bonds with three adjacent carbon atoms. The fourth valence electron in the unhybridized *p* orbital of each carbon atom is shared with two neighboring carbon atoms, forming π bonds transverse to the tube’s axis. On the other hand, hydrogen peroxide (H_2_O_2_), the simplest peroxide, consists of two hydrogen atoms and two oxygen atoms, and it is often used as an anti-infective agent. An oxygen atom has six valence electrons, and each of the hydrogen atoms has one electron to share. The oxygen atom is *sp^3^* hybridized. There is a covalent *sp^3^*-*sp^3^* bond between the two oxygen atoms. Each oxygen atom is covalently bonded to its respective hydrogen via *sp^3^*-*s* orbitals to form σ bonds. Remaining two lone-pair electrons on each oxygen atom is contained in the *sp^3^* hybridized orbitals. The optimized molecular structure of H_2_O_2_ is shown in Figure 2c and its bond lengths and bond angles are listed in Table 1, in accordance with literature data [75,76,77].

We first focused on the adsorption behavior of an H_2_O_2_ molecule on the pristine SWCNT. Because the H_2_O_2_ molecule tends to be adsorbed in various configurations, various input geometries should be considered. To this end, H_2_O_2_ can be placed above the C-C bond (A and Z sites), C atom (T site), and the center of the C hexagon (Ho site) of SWCNT, see Figure 2a. At these positions, the molecular axis could be aligned perpendicular or parallel with respect to the axis of the nanotube. By calculating the adsorption energy for different configurations of H_2_O_2_ on pristine SWCNT, the most favorable configuration was chosen. The vertical alignment of the hydrogen of the H_2_O_2_ on top of the C atom of the nanotube was discovered to be energetically more favorable, as shown in Figure 2d. The atomic radii are 0.67, 0.53, and 0.48 Å for C, H, and O atoms, respectively [78]. The minimum distance between the atoms of the H_2_O_2_ and the SWCNT (H-C) was found to be 1.9 Å, which is much larger than the sum of atomic radii of H and C (1.2 Å), indicating that the adsorption of H_2_O_2_ on pristine SWCNT is physical. The low adsorption energy (0.38 eV) and the small charge transfer from H_2_O_2_ to the nanotube (0.031 *e*) also suggest the interactions between H_2_O_2_ and SWCNT are relatively weak, governed by vdW forces. As a result, SWCNTs cannot capture peroxides.

**Figure 3:**
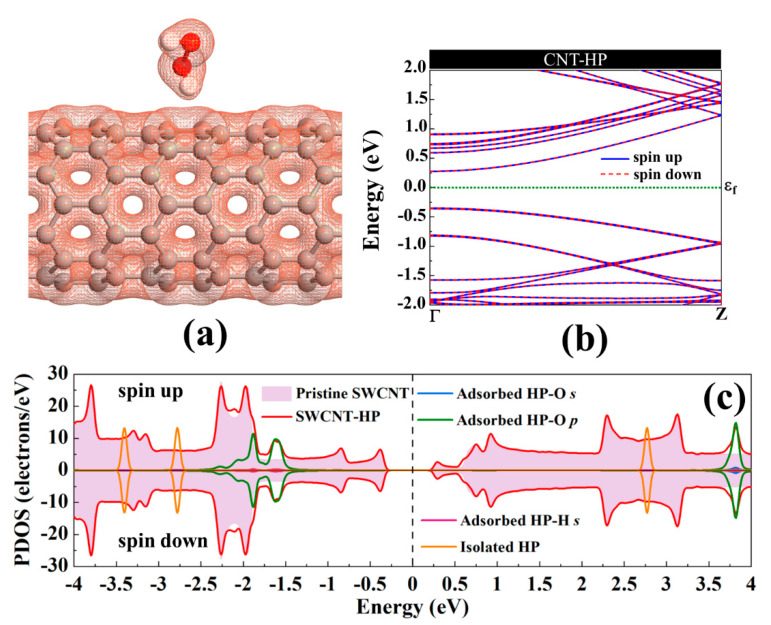
(**a**) The electronic total charge density, (**b**) energy band structure, and (**c**) total DOS curve for H_2_O_2_ adsorption on pristine (8,0) SWCNT. The Fermi level is set to zero.

Electron total charge density of the H_2_O_2_ adsorption on pristine SWCNT is shown in Figure 3a. As can be seen, because of the H_2_O_2_ physisorption on the nanotube, there is no orbital overlap between the H_2_O_2_ and the nanotube. Upon interaction, the structures of H_2_O_2_ and SWCNT were slightly changed. The diameter of the nanotube is enlarged (0.05 Å) in the interaction orientation (parallel to the axis of the nanotube) and contracted (0.05 Å) in the perpendicular direction. The C_a_-C_b_ of the nanotube is slightly reduced (0.01 Å) after interaction with H_2_O_2_ as a result of charge transfer. The band structure of SWCNT-H_2_O_2_ is provided in Figure 3b. The energy bandgap of SWCNT drops 0.016 eV upon interaction with H_2_O_2_, consistent with small charge transfer between the molecule and the nanotube. The H_2_O_2_-SWCNT is not a magnetic system. H_2_O_2_ has a slight impact on the total DOS (Density of States) near the Fermi level, as can be seen from Figure 3c. The H_2_O_2′_s highest occupied molecular orbital is placed deep in the valence band, and its lowest unoccupied molecular orbital is positioned high in the conduction band of the SWCNT. Hence, the semiconducting nature of SWCNT has been preserved after H_2_O_2_ adsorption.

**Figure 4:**
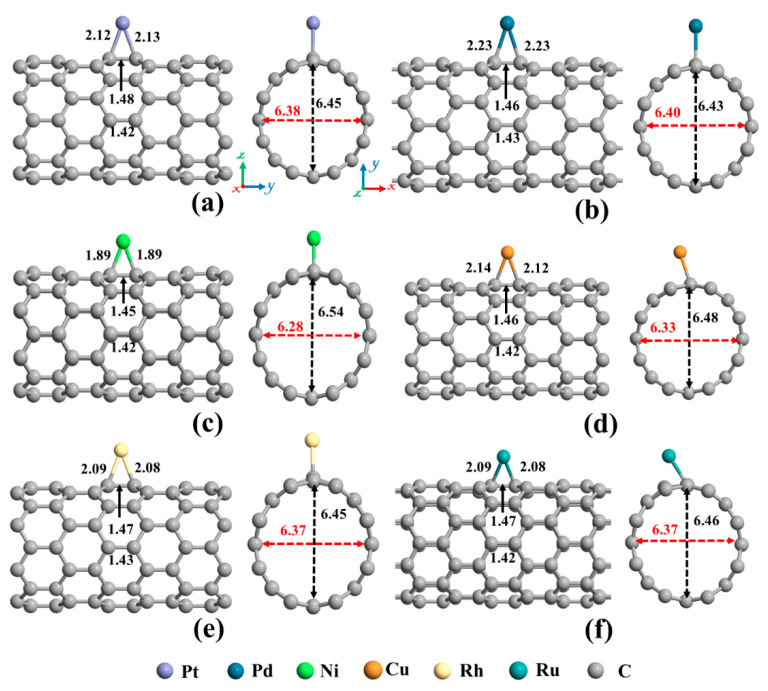
The optimized structures of a single (**a**) Pt, (**b**) Pd, (**c**) Ni, (**d**) Cu, (**e**) Rh, and (**f**) Ru decorated (8,0) SWCNT. The bond lengths, the diameters, and the binding distances between the metal atom and the nanotube are also given in the unit of Å. The sticks between atoms are only for visualization. The red and black dotted arrows present the diameter of the nanotube parallel and perpendicular orientation to the axis of the nanotube, respectively.

The next topic of our study is the decoration of the SWCNT with transition metal atoms (Pt, Pd, Ni, Cu, Rh, or Ru). From Figure 2a, three possible sites (Ho, T, and A) were considered for individual atom adsorption. After optimizing the structures and calculating the adsorption energies, the most favorable adsorption configurations were selected for further studies, as shown in Figure 4. The binding distance between the metal atom and the SWCNT, adsorption energy, the total magnetic moment, and charge transfer for these desired metals decorated SWCNTs are presented in Table 2. The results demonstrated that all the metals tend to be adsorbed on the A site and interact with two C atoms (C_a_ and C_b_, as labeled in Figure 2) of the SWCNT. The Pt-C_a_ (C_b_) distances are 2.12 (2.13) Å, Pd-C_a_ (C_b_) distances are 2.23 (2.23) Å, Ni-C_a_ (C_b_) distances are 1.89 (1.89) Å, Cu-C_a_ (C_b_) distances are 2.14 (2.12) Å, Rh-C_a_ (C_b_) distances are 2.09 (2.08) Å, and Ru-C_a_ (C_b_) distances are 2.09 (2.08) Å. The creation of metal-C bonds deteriorates the strength of C_a_-C_b_ bonds, causing these bonds to get enlarged. The C_a_-C_b_ bond length in the pristine SWCNT is elongated from 1.42 Å to 1.48, 1.46, 1.45, 1.46, 1.47, and 1.47 Å after decorating with Pt, Pd, Ni, Cu, Rh, and Ru atoms, respectively. The carbon atoms connected with metal atoms protrude a little outer of the tube, signifying the presence of *sp^3^* hybridization. As a result, the diameter of the decorated CNT is enlarged in the decoration orientation and contracted in the perpendicular direction. The values of diameter for the Pt-SWCNT, Pd-SWCNT, Ni-SWCNT, Cu-SWCNT, Rh-SWCNT, and Ru-SWCNT are 6.45 (6.38), 6.43 (6.39), 6.54 (6.28), 6.48 (6.33), 6.45 (6.37), and 6.46 (6.37) Å in the direction of the decoration (perpendicular to the decoration orientation), respectively. Clearly, the nanotube experienced its largest deformation upon interaction with the Ni atom. It agrees well with the strength of the Ni-C bond that has the shortest length among metal-C bonds. The atomic radii are 0.67, 1.77, 1.69, 1.49, 1.45, 1.73, and 1.78 Å for C, Pt, Pd, Ni, Cu, Rh, and Ru atoms, respectively [78]. The metal-C distances are smaller than the sums of C and metal atomic radii, showing that the metal atoms are chemically adsorbed on the nanotube.

The adsorption energy for Pt, Pd, Ni, Cu, Rh, and Ru on SWCNT were calculated to be −2.57, −1.53, −2.02, −0.75, −2.63, and −2.96 eV, respectively, in good agreement with literature data [64,68,74,79,80,81,82,83,84]. The interactions between the Pt, Pd, Ni, Cu, Rh, and Ru atoms and SWCNT produced a charge transfer of 0.159, 0.125, 0.502, 0.426, 0.312, and 0.376 *e* from the metals to the nanotube, respectively. One can notice that the higher adsorption energy does not necessarily lead to the larger charge transfer. A possible reason is that when the transition metals and C atoms of SWCNT interact, a donation [electron transfer from the bonding π (highest occupied molecular orbital)] states of the nanotube to the unoccupied orbitals of metal) and a back donation [electron transfer from the occupied orbitals of metal to anti-bonding π* (lowest unoccupied molecular orbital) states of the nanotube] occur [85]. Hence, the net charge transfer for metal and SWCNT interaction is a back donation. The values of electronegativity of C, Pt, Pd, Ni, Cu, Rh, and Ru atoms are 2.55, 2.28, 2.20, 1.91, 1.95, 2.28, and 2.20, respectively. Due to the higher electronegativity of C atoms compared to the transition metals, C atoms attract electrons from the decorated metal atom. Hereafter, the metal atoms are positively charged. Furthermore, the amounts of charge transfer are more pronounced in the case of Ni and Cu atoms as a result of higher electronegativity differences between these atoms and C atom.

**Figure 5:**
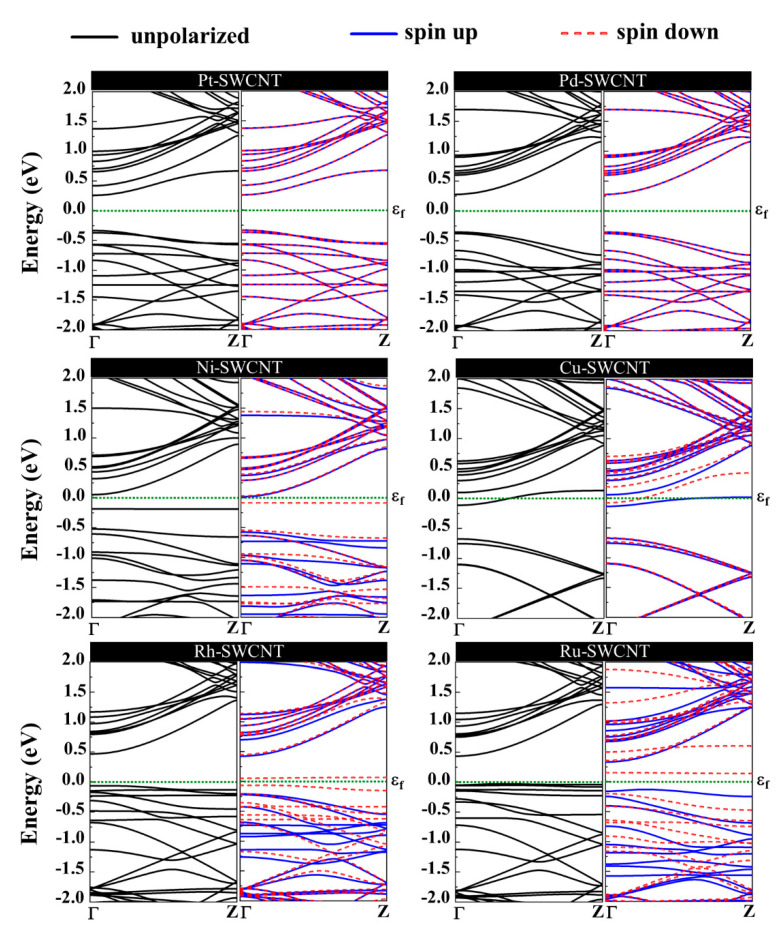
Energy band structures of Pt-SWCNT, Pd-SWCNT, Ni-SWCNT, Cu-SWCNT, Rh-SWCNT, and Ru-SWCNT systems. The dotted green line indicates the Fermi level, which is set to zero.

**Figure 6:**
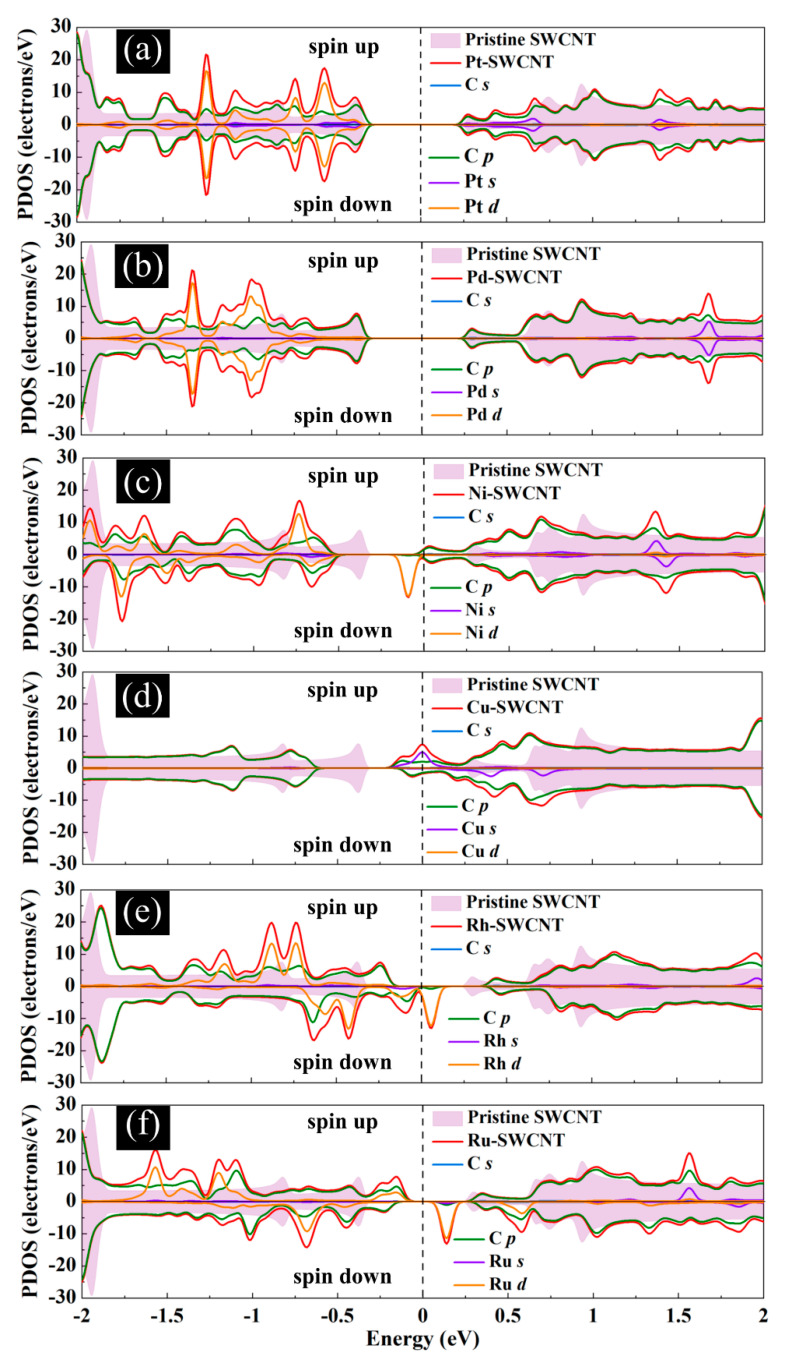
Total DOS curves for pristine SWCNT and (**a**) Pt-SWCNT, (**b**) Pd-SWCNT, (**c**) Ni-SWCNT, (**d**) Cu-SWCNT, (**e**) Rh-SWCNT, and (**f**) Ru-SWCNT systems. Projected density of states (PDOS) of the s and p orbitals of C atoms in SWCNT and s and d orbitals of metal atoms are also presented. The positive and negative values denote the spin-up and spin-down channels, respectively. The dashed lines indicate the Fermi level, which is set to zero.

The magnetic and electronic properties of the SWCNT after interaction with metal atoms undergo considerable changes. Energy band structures and the projected density of states (PDOS) of the metal decorated SWCNTs are presented in Figure 5 and Figure 6. While the Pt- and Pd-SWCNT systems have zero magnetic moments, decoration of SWCNT with Ni, Cu, Rh, or Ru atom introduces magnetism in the nanotube with the total magnetic moment of 0.28, 0.44, 0.90, and 1.74 µ_B_, respectively. The spin-polarized character of Ni-, Cu-, Rh-, and Ru-SWCNT systems and non-spin polarized character of Pt- and Pd-SWCNT systems can be corroborated by the band structures presented in Figure 5. The valence band and conduction band do not start at the same energy level in the spin-polarized systems, indicating their magnetic behavior. While the semiconducting properties of pristine SWCNT remain unchanged after decoration with Pt, Pd, N, Rh, or Ru with the corresponding bandgaps of 0.591, 0.630, 0.238, 0.531, or 0.463 eV, the SWCNT undergoes semiconductor-to-metal transition after interaction with Cu. The PDOS curves of spin-up and -down channels are symmetric in Pt-SWCNT and Pd-SWCNT systems while they are asymmetric in Ni-SWCNT, Cu-SWCNT, Rh-SWCNT, and Ru-SWCNT, indicating that Ni, Cu, Rh, or Ru impurity states induce magnetism in the nanotube. Except for Pt- and Pd-SWCNT systems, decoration of the nanotube with Ni, Cu, Rh, or Ru atoms introduce significant peaks in the bandgaps of the pristine SWCNT system near Fermi level The orbital hybridization between *d*-orbital of the metal atom and *p*-orbital of C atoms (π^*^ states) near Fermi level is responsible for strong interaction and the largest adsorption energy. While Ru-SWCNT shows the maximum orbitals overlap between Rh *d* and C *p* peaks, Cu-SWCNT does not present any significant overlap between the peaks, which are consistent with their corresponding adsorption energies.

The Brewer-Engel valence-bond theory explains the various strengths of metal bonds [85]. While the electrons of *s* and *p* orbitals of the transition metals describe the long-range structure, and the electrons of the *d* orbital determine the short-range bonding, accordingly the strength of a bond [86]. The transition metals studied here have the following electron configurations: Pt ([Xe] 4*f* ^14^ 5*d* ^9^ 6*s* ^1^), Pd ([Kr] 4*d* ^10^), Ni ([Ar] 3*d* ^8^ 4*s* ^2^), Cu ([Ar] 3*d* ^10^4*s* ^1^), Rh ([Kr] 4*d* ^8^ 5*s* ^1^), and Ru ([Kr] 4*d* ^7^ 5*s* ^1^). The *d*-bands of Pd and Pt atoms with 10 and nine electrons, respectively, are almost full. However, the orbital diffusiveness for the 5*d* orbitals of Pt is stronger than that for the 4*d* orbital of Pd. Thus, the orbital overlap between the π electron cloud of C atoms and *d* orbital of the Pt atom is stronger than the Pd atom. Ni and Rh have eight electrons in their corresponding *d*-bands, signifying that their *d* orbitals have more tendency for hybridization with the π electron cloud of C atoms. Ru, with seven electrons in its *d*-bands, has more vacant contributions than other metals, resulting in a maximal overlap between *d* orbitals and π electron cloud. By the same token, Cu has a nearly full *d*-band. Nevertheless, the 3*d* orbitals of the Cu are less diffuse than 4*d* and 5*d* orbitals, making the least overlap between π electron cloud of C atoms and *d* orbital of the considered metals. These results are consistent with the order of adsorption energies listed in Table 1.

**Figure 7:**
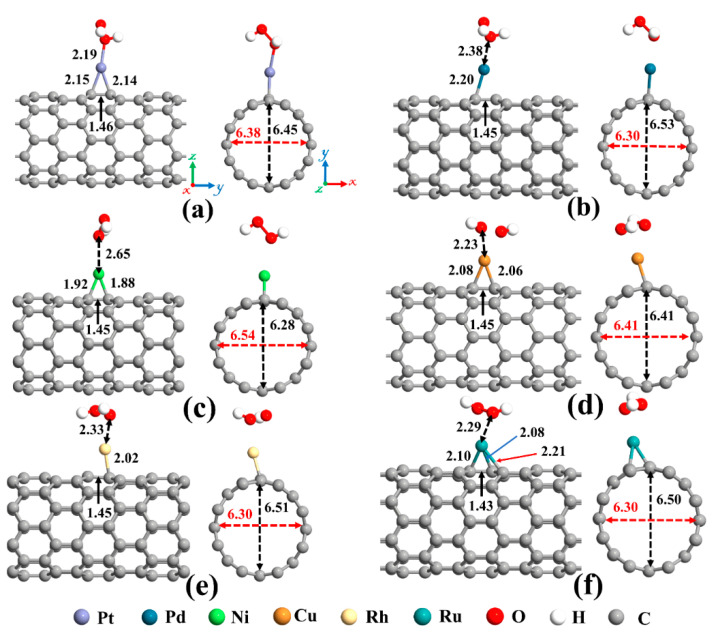
Optimized structures of H_2_O_2_ on (**a**) Pt-SWCNT, (**b**) Pd-SWCNT, (**c**) Ni-SWCNT, (**d**) Cu-SWCNT, (**e**) Rh-SWCNT, and (**f**) Ru-SWCNT systems. The bond lengths, the diameters, and the binding distances between the metal atom and the nanotube are also given in the unit of Å. The sticks between atoms are only for visualization. The red and black dotted arrows present the diameter of the nanotube parallel and perpendicular orientation to the axis of the nanotube, respectively.

Eventually, adsorption of H_2_O_2_ on metal decorated SWCNTs was studied, and the most stable structures are represented in Figure 7. In all cases, the H_2_O_2_ molecule prefers its O atom to be close to the metal atom. Except for the Cu-SWCNT that dissociates H_2_O_2_ into two OH species, the H_2_O_2_ retains its molecular form over other metal decorated SWCNT systems. The O-O bond in H_2_O_2_ after interaction with metal decorated SWCNTs is elongated by 4.7, 1.3, 3.4, 39.7, and 2.05% for Pt, Pd, Ni, Cu, and Rh, respectively. However, the O-O bond for Ru-SWCNT is shortened by 2.7%. The H-O-O bond angle of the H_2_O_2_ is decreased from 101.02° for an isolated H_2_O_2_ to 99.3° for Pt-SWCNT, 99.7° for Pd-SWCNT, 99.8° for Ni-SWCNT, 101.3° for Ru-SWCNT, respectively, but is increased for Cu- and Rh-SWCNT to 113.6° and 102.7°, respectively.

As mentioned before, upon interaction with SWCNT, the metal atoms are charged positively, while the nanotube becomes rich in electrons. Henceforward, metal atoms work as the capturing center for the H_2_O_2_ that cannot be trapped by the pristine SWCNT. The distance between Pt, Pd, Ni, Cu, Rh, and Ru atoms and C_a_ (C_b_) (as labeled in Figure 2a) of CNT are 2.15 (2.14), 2.20 (2.28), 1.92 (1.88), 2.08 (2.06), 2.23 (2.02), and 2.10 (2.08) Å, respectively, after interaction with H_2_O_2_ molecule. Moreover, the Ru atom is slightly moved toward the hollow position (the center of the hexagon ring). The Ru-C_c_ and Ru-C_d_ distances are 2.25 Å and 2.21 Å, correspondingly. Due to the *sp^3^* hybridization, the C atoms of the nanotube in the interaction area are slightly protruded. This caused the diameter of the Pd-, Rh-, and Ru-SWCNT to be enlarged in the decoration direction. The diameter of Pt-SWCNT does not change, and the diameter of Ni- and Cu-SWCNT is shortened in the decoration direction. The values of the diameter for the Pt-SWCNT, Pd-SWCNT, Ni-SWCNT, Cu-SWCNT, Rh-SWCNT, and Ru-SWCNT systems after interaction with H_2_O_2_ are 6.45 (6.38), 6.53 (6.30), 6.28 (6.54), 6.41 (6.41), 6.51 (6.30), and 6.50 (6.30) Å in the orientation of the adsorbed metal (perpendicular to the adsorption orientation), respectively. It is worth mentioning that the C_a_-C_b_ bond is somewhat augmented upon the interaction of H_2_O_2_ with metal decorated SWCNT because of deterioration of the strength of metal-C bonds. The C_a_-C_b_ bond length in the Pt-, Pd-, Ni-, Cu-, Rh-, and Ru-SWCNT changed from 1.48, 1.46, 1.45, 1.46, 1.47, and 1.47 Å to 1.46, 1.45, 1.45, 1.45, 1.45, and 1.43 Å upon H_2_O_2_ adsorption, respectively.

**Figure 8:**
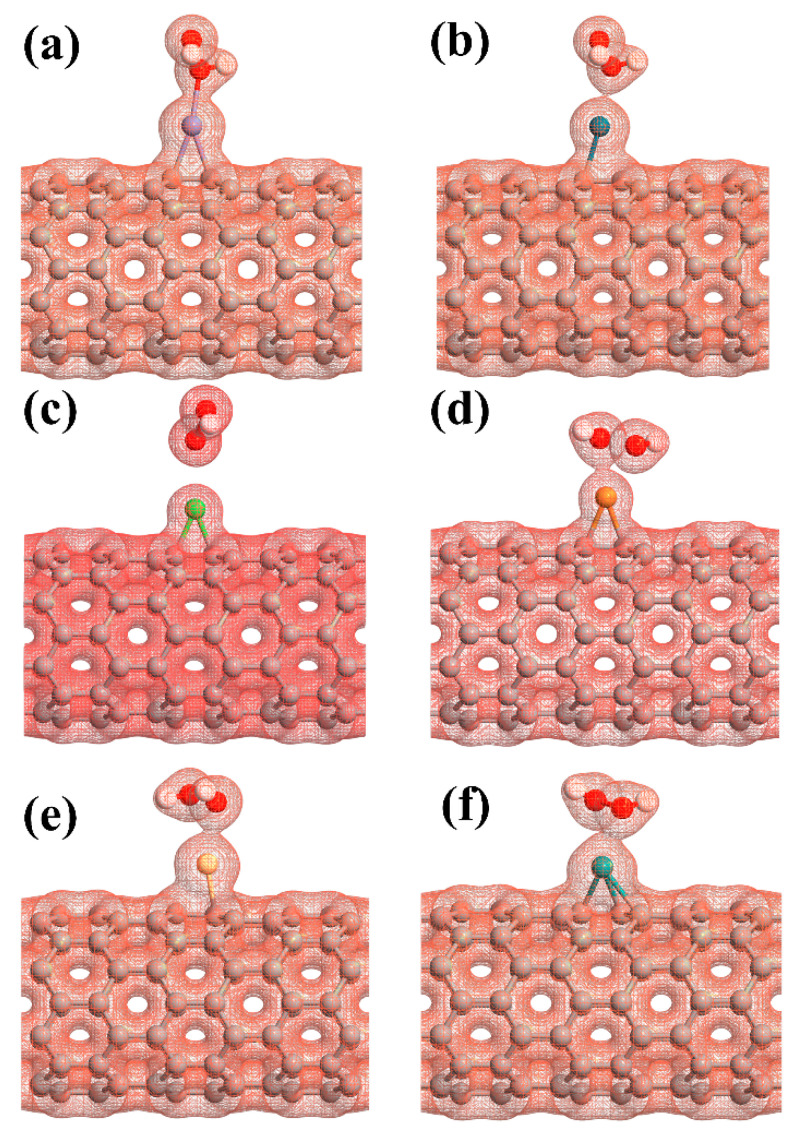
The electronic total charge densities for the adsorption of H_2_O_2_ on (**a**) Pt-SWCNT, (**b**) Pd-SWCNT, (**c**) Ni-SWCNT, (**d**) Cu-SWCNT, (**e**) Rh-SWCNT, and (**f**) Ru-SWCNT systems. The sticks between atoms are only for visualization.

The adsorption energy, binding distance, magnetic moment, charge transfer, energy bandgap, and recovery time of H_2_O_2_-metal-SWCNTs are presented in Table 3. The total charge transfer from H_2_O_2_ to Pt-, Pd-, Ni-, Cu-, Rh-, and Ru-SWCNTs were also found to be 0.108, 0.091, 0.056, 0.298, 0.105, and 0.218 e, respectively. The electrons are transferred from the H_2_O_2_ to the C atoms of SWCNT in the interaction area through metal atoms. The adsorption energies were calculated to be −2.12, −1.14, −1.25, −1.88, −1.17, and −1.23 eV for H_2_O_2_ adsorption on Pt-, Pd-, Ni-, Cu-, Rh-, and Ru-decorated SWCNT systems, respectively. The atomic radii are 0.48, 1.77, 1.69, 1.49, 1.45, 1.73, and 1.78 Å for O, Pt, Pd, Ni, Cu, Rh, and Ru atoms, respectively [78]. The sums of O and Pt, Pt, Pd, Ni, Cu, Rh, and Ru atomic radii are 2.25, 2.17, 1.97, 1.93, 2.21, and 2.26 Å, respectively. Based on our calculations, the O-Pt, O-Pd, O-Ni, O-Cu, O-Rh, and O-Ru distances were found to be 2.19, 2.38, 2.65, 2.23, 2.33, and 2.29 Å, respectively. It can be deduced that the O-Pt bond length is less than the sum of corresponding atomic radii, implying that the bond between H_2_O_2_ and Pt-SWCNT is covalent. For the rest of the cases, the O-Metal bond length is larger than the sum of corresponding atomic radii. To gain more insight into the adsorption of H_2_O_2_ on metal decorated SWCNTs, their electronic total charge densities were calculated and presented in Figure 8. In the case of Pt-SWCNT-H_2_O_2_ system, a strong orbital overlap is observed between Pt and O atom of H_2_O_2_, confirming the strong chemisorption. For Pd-CNT-HP, a weak orbital mixing between Pd and O atom is noticeable, suggesting weak chemisorption. There is no orbital overlap between H_2_O_2_ and Ni-SWCNT systems. Considering the adsorption energy (−1.25 eV), the interaction between H_2_O_2_ and Ni-SWCNT could be strong physisorption. A moderate orbital mixing between Cu and O atom of radical OH is observed in the Cu-SWCNT-H_2_O_2_ case. In the case of Rh- and Ru-CNT- H_2_O_2_, Rh and Ru atoms have moderate orbital overlap with O atom of H_2_O_2_, presenting moderate chemisorption.

**Figure 9:**
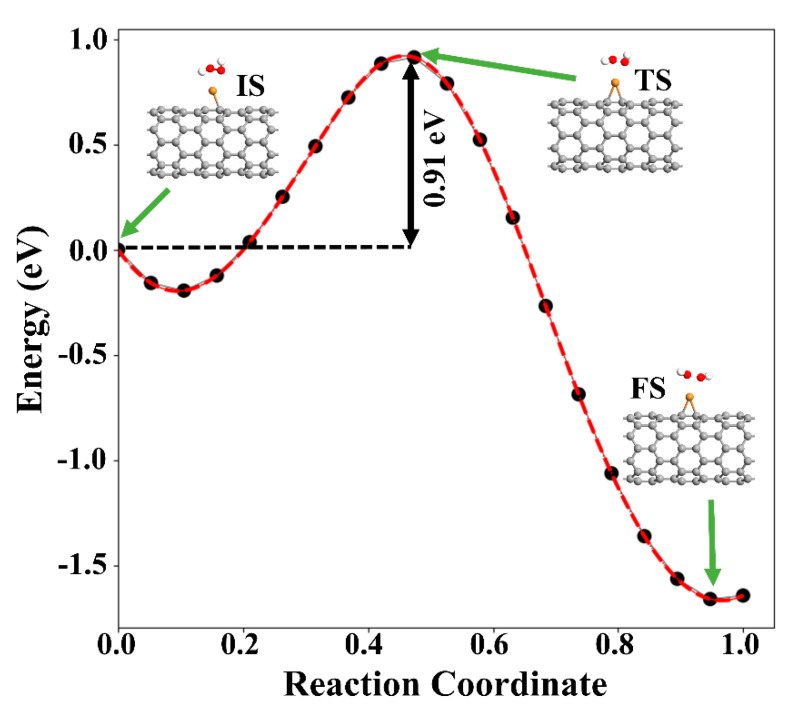
MEP of H_2_O_2_ decomposition hydroxyl radicals (OH) on the Cu-SWCNT. The initial state (IS), transition state (TS), and final state (FS) are shown in the inset. The green arrows show the initial, transition, and final states.

Next, the dissociation of the H_2_O_2_ molecule into two OH species over Cu-SWCNT was examined. As stated above, the reaction is exothermic, with an adsorption energy of −1.88 eV. The increase of the O−O bond distance from 1.46 to 2.04 Å supports the dissociation of H_2_O_2_, which is accepted as the rate-determining step for the H_2_O_2_ reduction reaction [49]. The O atoms of two OH species are placed at 2.23 and 2.44 Å from Cu. To investigate the dissociation proves of H_2_O_2_, climbing image nudged (NEB) was performed. Figure 9 presents the minimum energy path (MEP). The energy barrier associated with the dissociation of H_2_O_2_ into two OH radicals was found to be 0.91 eV. This suggests that the dissociation of H_2_O_2_ is likely to happen on the Cu-SWCNT surface.

**Figure 10:**
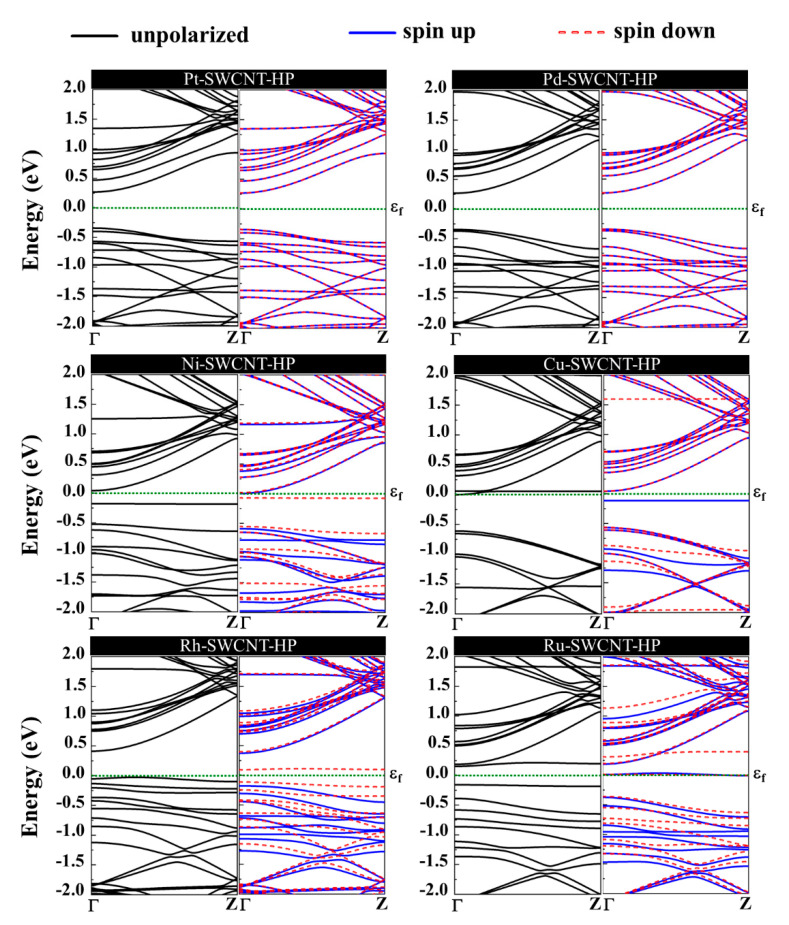
Energy band structures of H_2_O_2_ adsorption on Pt-SWCNT, Pd-SWCNT, Ni-SWCNT, Cu-SWCNT, Rh-SWCNT, and Ru-SWCNT systems. The dotted green line indicates the Fermi level, which is set to zero.

The orbital hybridization and the charge transfer from H_2_O_2_ to metal-SWCNTs cause significant changes to the magnetic and electronic properties of the nanotubes. Figure 10 presents the energy band structure of metal-SWCNTs after H_2_O_2_ adsorption. The energy bandgap of Pt-SWCNT (Pd-SWCNT) increases (decreases) from 0.591 (0.630) eV to 0.611 (0.610) eV after interaction with H_2_O_2_, indicating that the conductivity of the system decreases (increases). The Pt- and Pd-CNT- H_2_O_2_ systems show no magnetic properties. For Ni-, Cu-, Rh, and Ru-SWCNT systems, which are spin-polarized before gas molecule adsorption, they remain spin-polarized after interaction with H_2_O_2_ molecule with a total magnetic moment of 0.350, 0.790, 0.886, and 0.966 µ_B_, respectively. The H_2_O_2_ dissociation does not change the metallic behavior of Cu-SWCNT. Besides that, the energy bandgap of Ni-, Rh-, and Ru-SWCNT drops from 0.238, 0.531, and 0.463 eV to 0.220, 0.439, and 0.309 eV, respectively, upon H_2_O_2_ adsorption.

**Figure 11:**
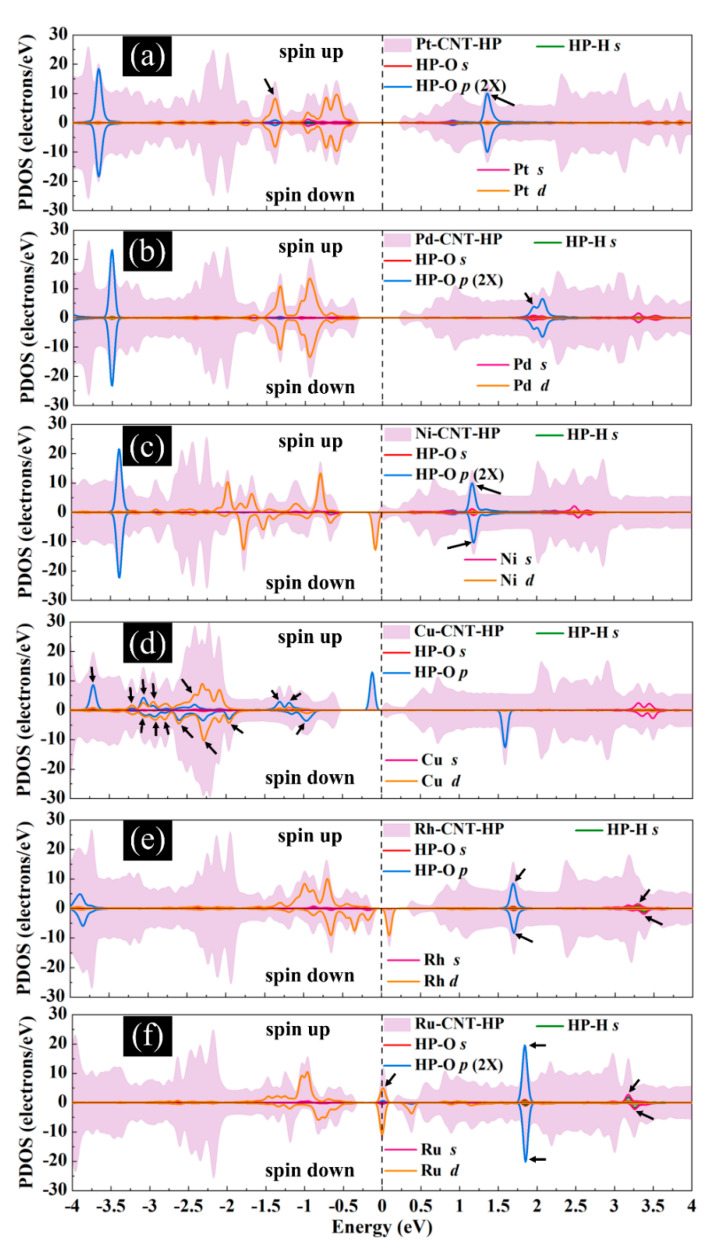
Total DOS curves for H_2_O_2_ adsorption on (**a**) Pt-SWCNT, (**b**) Pd-SWCNT, (**c**) Ni-SWCNT, (**d**) Cu-SWCNT, (**e**) Rh-SWCNT, and (**f**) Ru-SWCNT systems. PDOS of the s and p orbitals of O atoms and s orbital of H atoms in H_2_O_2_ and s and d orbitals of metal atoms are also presented. The positive and negative values denote the spin-up and spin-down channels, respectively. The dashed lines indicate the Fermi level, which is set to zero. The black arrows present the orbital hybridization between metal atoms and the H_2_O_2_ molecule.

Figure 11 presents the DOS curves of metal-SWCNT-H_2_O_2_ systems. The asymmetric PDOS distribution of Ni-, Cu-, Rh, and Ru-SWCNT systems indicate that these systems show magnetic behavior. The hybridization between the orbitals of the metal atom and the orbitals of O makes the main contribution to the metal-SWCNT-H_2_O_2_ interaction. The orbital hybridization occurs when the peaks of two orbitals lie in the same energy. In the case of Pt-CNT-H_2_O_2_, two overlapping peaks (one strong and one weak) between Pt *d* orbital and O *p* orbital were observed. One weak overlapping peak between Pd *s* orbital and O *p* orbital was also found for Pd-CNT-H_2_O_2_. This suggests a stronger hybridized interaction between H_2_O_2_ and Pt-SWCNT in comparison with H_2_O_2_ and Pd-SWCNT. Similar to the Pd-SWCNT-H_2_O_2_ system, the PDOS of the Ni-CNT-H_2_O_2_ system presents one overlapping peak between Ni *s* orbital and O *p* orbital. The coupling peaks between the two OH species and Cu atom are widely distributed. Seven overlapping peaks between Cu *d* orbital and O *p* orbital were discovered. The dissociative adsorption of H_2_O_2_ on Cu-SWCNT introduces a significant spin-up peak close to the Fermi level in the valence band, which may be the cause of the high adsorption energy. Rh-SWCNT-H_2_O_2_ indicates two overlapping points, one between Rh *s* orbital and O *p* orbital and one between Rh *s* orbital and H *s* orbital. For the Ru-SWCNT-H_2_O_2_ system, three overlapping peaks were detected, one between Ru *d* orbital and O *p* orbital, one between Ru *s* orbital and O *p* orbital, and one between Ru *s* orbital and H *s* orbital. Interestingly, compared to Figure 6f, the peak associated with Ru appeared at the Fermi level. One possible reason is that Ru atom adsorbed on the SWCNT is slightly shifted toward the center of the hexagon ring after interaction with H_2_O_2_.

In order to develop a reliable viral capture and inactivation surface, it is crucial to ensure that H_2_O_2_ can be captured and retained by the CNT surface for a very long time. Table 3 lists the recovery time of the considered systems. The recovery time (τ) can be expressed using the conventional transition state theory as follows [87]:

(1) $$\tau =\upsilon_{{}_{0}}^{-1}\exp\;(-E_{ad}/k_{B}T)$$

where $\upsilon_{0}$ is the attempt frequency, T is the temperature, and k_B_ is the Boltzmann constant. It is expected that at a constant temperature, small adsorption energies result in a fast desorption process of the H_2_O_2_ gas. The obtained recovery times for H_2_O_2_ desorption from pristine SWNCT, Pt-, Pd-, Ni-, Cu-, Rh-, and Ru-CNT systems at room temperature under UV radiation ($\upsilon_{0}=10^{16}\;Hz$) are 2.6 × 10^−10^, 6.9 × 10^19^, 1.8 × 10^3^, 1.3 × 10^5^, 6.1 × 10^15^, 6.1 × 10^3^, and 6.2 × 10^4^ sec, respectively. The long recovery time obtained for Pt-SWCNT and Cu-SWCNT after interaction with H_2_O_2_ (2.2 × 10^12^ and 1.9 × 10^8^ years, respectively) suggests that Pt- and Cu-decorated SWNCT-H_2_O_2_ systems have an excellent potential for virus removal filters with a very long shelf-life.

From Equation (1), one can deduce that the recovery time can be shortened by enhancing the temperature at constant adsorption energy. The recovery times for H_2_O_2_ desorption from Pt-, Pd-, Ni-, Cu-, Rh-, and Ru-CNT systems at T = 398 K under UV radiation ($\upsilon_{0}=10^{16}\;Hz$) are 5.0 × 10^10^, 0.02, 0.55, 4.7 × 10^7^, 0.05, and 0.31 sec, respectively. Hence, Pd-, Ni-, Rh-, and Ru-SWCNT systems could be promising candidates for H_2_O_2_ detection and detection of viral capture based on the H_2_O_2_ signal ascribable to their fast recovery. The conductivity change of material by gas adsorption is a good predictor for the detection of viruses and their inactivation. The sensitivity (S), which is the variation of the conductivity (σ) for the nanosensors with and without viruses, is expressed as [88]:

(2) $$S=\frac{\sigma_{\mathrm{virus}}-\sigma_{H_{2}O_{2}}}{\sigma_{\mathrm{virus}}}\times 100$$

where σ_Virus_ and σ_H2O2_ are the conductivity of the system after and before viral capture, respectively. The electrical conductivity of a material can be determined as [88]:

(3) $$\sigma \propto \exp\;(-E_{g}/2k_{B}T)$$

where E_g_ is the bandgap, T is the temperature, and k_B_ is the Boltzmann constant. Pristine SWCNT shows 37.71% sensitivity toward H_2_O_2_. It was also found that the sensitivities of Pt-, Pd-, Ni-, Rh-, and Ru-SWCNT systems for H_2_O_2_ detection are 32.96%, 49.18%, 13.33%, 529.65%, and 2075.84%, respectively. Exceptional sensitivities obtained for Rh-SWCNT and Ru-SWCNT systems accompanied by very fast recovery introduce these systems as potential compositions for detection, capture, and viral inactivation, all of which can be accomplished by using nanotube-based molecular sensors.

## 3. Discussion

The high mortality due to SARS-CoV-2 and other viral infections can be attributed to unmitigated biological transmission pathways and to the fact that a number of viruses can remain infectious for days on varying surfaces. Both airborne and hospital fomites present a significant path for viral transmission. Past work based on long-range airborne, fomite, and combined routes in 1744 scenarios have shown that the combined route of long-range airborne and fomites present significant biological transmission pathways [89]. Mitigating these transmission pathways can provide better environmental hygiene for hospital staff and patients, as research suggests that a large number of respiratory illnesses can be prevented through improved cleaning of surfaces [90]. The use of chemicals for cleaning presents an additional challenge due to long exposure to some chemicals that can also be detrimental to human health. A concentration of 3% H_2_O_2_ in water kills most of the enveloped viruses on conventional surfaces. Furthermore, molecular H_2_O_2_ has been recommended to be a safe and efficacious way of removing potentially virus-contaminated objects from biocontainment level III laboratories in which exotic animal disease virus agents are handled [91]. The use of nanomaterial surfaces can enable instant inactivation of viruses due to the intrinsic antiviral properties of nanomaterials. The use of ROS promoting agents such as H_2_O_2_ in a molecular form on nanosurfaces potentially brings the viruses close to the H_2_O_2_ electron cloud, thereby exchanging electrons with the lipid membrane, resulting in its break down. This field is still in its infancy as currently, there is no understanding of how even a virus attaches to different nanomaterial surfaces, including entropic and free energy considerations.

We investigated the types of materials that would promote ROS and have a long shelf-life on SWCNT surfaces using DFT based methods. To our surprise, a number of metals, including Pt, Pd, Ni, Cu, Rh, and Ru, promote the capture of H_2_O_2_ on SWCNT surfaces compared to just bare SWCNTs. Our theoretical study opens new avenues of creating viral inactivation nanosurfaces based on SWCNT-metal nanocomposites. While the DFT study was based on individual atoms on single SWCNTs, nanoparticles of such metals (1-5 nm) can be highly reactive on SWCNT surfaces towards viral capture and killing. The long adsorption of H_2_O_2_, especially on Pt and Cu based SWCNT molecular nanocomposites suggest a long shelf-life. Our group has created free-standing SWCNT membranes (both randomly oriented and highly aligned liquid crystals) that can be handled by hand, cut into any shapes and sizes [71,72,73]. Such membranes and large area surfaces integrated with nanoparticles of metals can have a long shelf-life for peroxide adsorption and can act as an effective barrier for viral transmission. These surfaces can also be custom assembled on hospital elastomeric and other fomites, into textiles for masks, and in air-filters. Our theoretical study thus presents an important advancement towards informing experiments on the types of nanomaterials that can be used to fight against viruses. SWCNTs, with their high mechanical strength, flexibility, ability to create a large surface area, ability to tailor the surface with metal nanoparticles, and long-time scales for H_2_O_2_ adsorption on SWCNT nanocomposites, present tantalizing opportunities for creating self-sanitizing surfaces.

## 4. Materials and Methods

This study presents first-principles calculations based on DFT, implemented in Atomistix ToolKit (ATK) software package [20,92,93]. Spin-polarized Generalized Gradient Approximation of Perdew-Burke-Ernzerhof (GGA-PBE) exchange-correlation functional with the Hartwigsen, Goedecker, Hutter (HGH) pseudopotentials with tier 3 was adopted. The Grimme van der Waals (vdW) correction (PBE-D2) [94] was also engaged to take into account the long-range vdW interactions [95]. The electronic temperature was 300 °K, and the plane wave mesh cut-off energy was 150 Rydberg.

A zigzag (8,0) SWCNT was considered, consisting of 96 C atoms in a periodically repeated supercell with lattice constants of a = b = 30 Å, large enough to avoid spurious image-image interactions, and c = 12.82 Å. The average C-C bond length and diameter of the nanotube were found to be 1.42 Å and 6.40 Å, respectively. In order to study the metal-SWCNT structure, a metal atom was added to the SWCNT supercell. By employing the conjugate gradient method, all the structures were allowed to fully relax until the final atomic force on each atom is less than 0.01 eV/Å. The Brillouin zone was sampled with a 1 × 1 × 11 Monkhorst-Pack *k*-point grid during geometry optimization. The *k*-point grid was then increased to 1 × 1 × 101 for electronic structure calculations to achieve more accurate results for electronic structure calculations. The adsorption energy provides quantitative data on the interaction strength between adsorbent and adsorbate. The vdW corrected adsorption energy of an H_2_O_2_ molecule on the pristine and metal decorated SWCNT is calculated by:

(4) $$E_{ad}=E_{M+X}-(E_{M}+E_{X})$$

where X is SWCNT or metal-SWCNT. E_M+X_, E_X_, and E_M_ are the total energies of the H_2_O_2-_nanotube complex, nanotube, and the isolated H_2_O_2_, respectively. A negative E_ad_ suggests that the adsorption process of H_2_O_2_ is exothermic, and the more negative value relates to a stronger interaction between the adsorbate and nanotube. Moreover, the charge transfer (Q_T_) upon adsorption of the H_2_O_2_ on the nanotube was obtained using Mulliken population analysis from counting the charge difference between the adsorbed and the isolated H_2_O_2_ molecule. A negative Q_T_ indicates a charge transfer from H_2_O_2_ to the nanotube, whereas a positive Q_T_ shows that H_2_O_2_ withdraws electrons from nanotube.

## 5. Conclusions

Surfaces capable of detecting, inactivating, and removal of viruses from the air or on fomites are vital to prevent viral transmission, especially in pandemics such as the one caused by SARS-CoV-2. For this purpose, we proposed H_2_O_2_ (as a representative of the ROS family) combined with metal decorated SWCNTs as an effective platform to fight against viruses. We utilized first-principles methods based on DFT to scrutinize the interaction behavior of H_2_O_2_ on metal (Pt, Pd, Ni, Cu, Rh, and Ru) decorated SWCNTs. Our aim of this study was to investigate SWCNT-metal systems as nano filter materials, PPEs and viral detection devices for future applications in detection, removal, and inactivation of viruses. The most stable adsorption configurations, adsorption energies, charge transfer, and electronic and magnetic properties of the H_2_O_2_ on the metal-nanotube were calculated. Our results indicated that Pt- and Cu-decorated SWCNT have an extraordinary capacity for H_2_O_2_ and OH radical capture with very long recovery time (2.2 × 10^12^ and 1.9 × 10^8^ years, respectively) compared to Pd (Results), Ni (Results), Rh (Result), and Ru (Result); however, these results suggest that SWNCT show potential as a largely previously unexplored substrate for capture, inactivation, and control of viral infectious diseases. Moreover, the exceptional sensitivities obtained for Rh-SWCNT (529.65%) and Ru-SWCNT (2075.84%) systems along with speedy recovery (milliseconds) suggest elastomeric fomites with integrated SWCNT-Ru/Rh systems as potential nanocomposites for self-cleaning antiviral surfaces such as PPE. Elastomeric respirators are being proposed for use in hospitals during pandemics. However, such respirators can also be viral transmission surfaces. The use of nanocomposites based on SWCNT-Ru/Rh-elastomeric systems with controlled diameters of nanoparticles (1–10 nm) functionalized with 3% H_2_O_2_ could potentially be useful as self-cleaning respirators and mitigate viral transmission on contact. Our results also call for the development of viral detection devices based on metal-decorated SWCNT systems. For example, a field-effect transistor based on SWCNT can be proposed with H_2_O_2_ functionalized surface to electrically determine the capture and inactivation of viruses using conductivity measurements and advanced classification methodologies. Such sensor networks could be integrated into the hospital’s working environment and to ensure the safety of medical personnel beyond a certain threshold. These sensors could also be integrated with smartphones that can potentially be used for personal safety monitoring. The similar size scale of viruses and SWCNT bundles and metal nanoparticles (<100 nm) suggest highly sensitive molecular sensors, and viruses capture agents and inactivation based on SWCNT-Rh/Ru-H_2_O_2_ based systems. While this field is in its infancy, due to the COVID-19 pandemic, there is an immediate urgency in the development of devices for viral detection, elastomeric nanocomposites based on SWCNT systems for PPE’s that can deactivate the viruses on contact. Our theoretical understanding of how SWCNT-metal systems interact with H_2_O_2_ show a new pathway for design of such antiviral surfaces based on controlling the SWCNT bundle diameters to <10–100 nm in nanocomposites and metal nanoparticles in 1–5 nm diameter range to be highly effective for H_2_O_2_ adsorption with long shelf-life for viruses inactivation and these results presented here could potentially drive future experiments based on the theoretical understanding. Such systems, if developed, could ensure the safety of health care workers in future pandemics.

## Figures and Tables

**Table 1:** Bond distances (d) and angles (∠) of the H_2_O_2_ (HP) molecule before and after interaction with pristine and metal decorated SWCNT.

Description	HP—Isolated	HP—SWCNT	HP—Pt-SWCNT	HP—Pd-SWCNT	HP—Ni-SWCNT	HP—Cu-SWCNT	HP—Rh-SWCNT	HP—Ru-SWCNT
d O-O	1.46	1.52	1.53	1.48	1.51	2.04	1.49	1.42
d O-H	0.99	0.99	1.00	1.00	1.00	1.00	1.00	1.00
∠ H-O-O	101.02	99.3	99.3	99.7	99.8	113.61	102.7	101.3

distances (d) in Å, angles (∠) in degrees.

**Table 2:** The calculated adsorption energy (E_ad_), the binding distance between the metal atom and the nanotube (D), magnetic moment (m), charge transfer (Q), energy bandgap (E_g_) for the spin-up (-down) channel. The negative values of charge indicate a charge transfer from metal to the nanotube. a: C_a_ atom and b: C_b_ atom in Figure 2a.

System	E_ad_ (eV)	D (Å)	m(µ_B_)	Q (*e*)	E_g_ (eV)
SWCNT	-	-	0.00	-	0.643
Pt-SWCNT	−2.57 (−2.604 [64], −2.7 [79])	2.12 (a) and 2.13 (b)	0.00	−0.159	0.591
Pd-SWCNT	−1.53 (−1.615 [64], −1.7 [79])	2.23 (a) and 2.23 (b)	0.00	−0.125	0.630
Ni-SWCNT	−2.02 (−2.4 [79])	1.89 (a) and 1.89 (b)	0.28	−0.502	0.238
Cu-SWCNT	−0.75 (−0.7 [79])	2.14 (a) and 2.12 (b)	0.44	−0.426	0.00
Rh-SWCNT	−2.63 (−2.67 [74])	2.09 (a) and 2.08 (b)	0.90	−0.312	0.531
Ru-SWCNT	−2.96 (−2.133 [84])	2.09 (a) and 2.08 (b)	1.74	−0.376	0.463

-: indicate not applicable.

**Table 3:** The calculated adsorption energy (E_ad_), binding distance (D), which is the distance between H_2_O_2_ molecule and the metal in the metal-decorated CNT structure or the shortest distance between the H_2_O_2_ molecule and the nanotube in the pristine CNT structure, magnetic moment (m), charge transfer (Q), energy bandgap (E_g_), and recovery time (τ) at T = 298 and 398 K under UV. The negative values of charge indicate a charge transfer from the molecule to the nanotube.

System	E_ad_ (eV)	D (Å)	m (µ_B_)	Q (*e*)	E_g_ (eV)	τ (sec) @ T = 298 K	τ (sec) @ T = 398 K	|S| (%)
CNT- H_2_O_2_	−0.38	1.9	0.00	−0.031	0.627	2.6 × 10^−10^	6.1 × 10^−12^	37.71
Pt-CNT- H_2_O_2_	−2.12	2.19	0.00	−0.108	0.611	6.9 × 10^19^	5.0 × 10^10^	32.96
Pd-CNT- H_2_O_2_	−1.14	2.38	0.00	−0.091	0.610	1.8 × 10^3^	0.02	49.18
Ni-CNT- H_2_O_2_	−1.25	2.65	0.350	−0.056	0.220	1.3 × 10^5^	0.55	13.33
Cu-CNT- H_2_O_2_	−1.88	2.23	0.790	−0.298	0.000	6.1 × 10^15^	4.7 × 10^7^	-
Rh-CNT- H_2_O_2_	−1.17	2.33	0.886	−0.105	0.439	6.1 × 10^3^	0.05	529.65
Ru-CNT- H_2_O_2_	−1.23	2.29	0.966	−0.218	0.309	6.2 × 10^4^	0.31	2075.84
